# Supplementary material for: Optimization of Callus Induction and Shoot Regeneration from Tomato Cotyledon Explants
Source: Plants (Basel). 2023 Aug 14;12(16):2942. doi: 10.3390/plants12162942 (PMC10459365; doi:10.3390/plants12162942)
Supplement: Supplementary file 1 [file plants-12-02942-s001.zip › Figure S1_to_S4.pdf]

Supplemental Figure S1

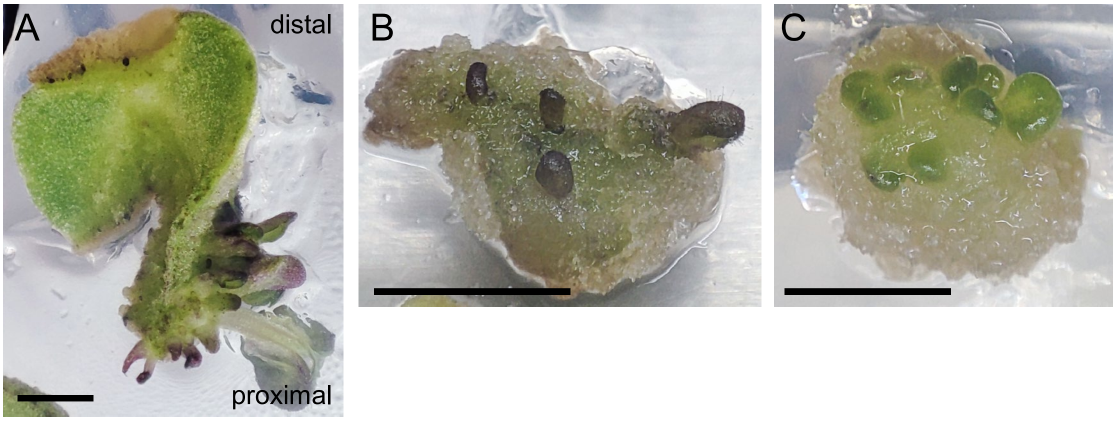

Suppl. Fig. S1. Hormone-induced shoot formation in M82 and MT tomato cultivars (cont.). Representative images of shoot formation in M82 (A, B) and MT (C) tomato cotyledon explants. Scale bars: 3 mm.

Supplemental Figure S2

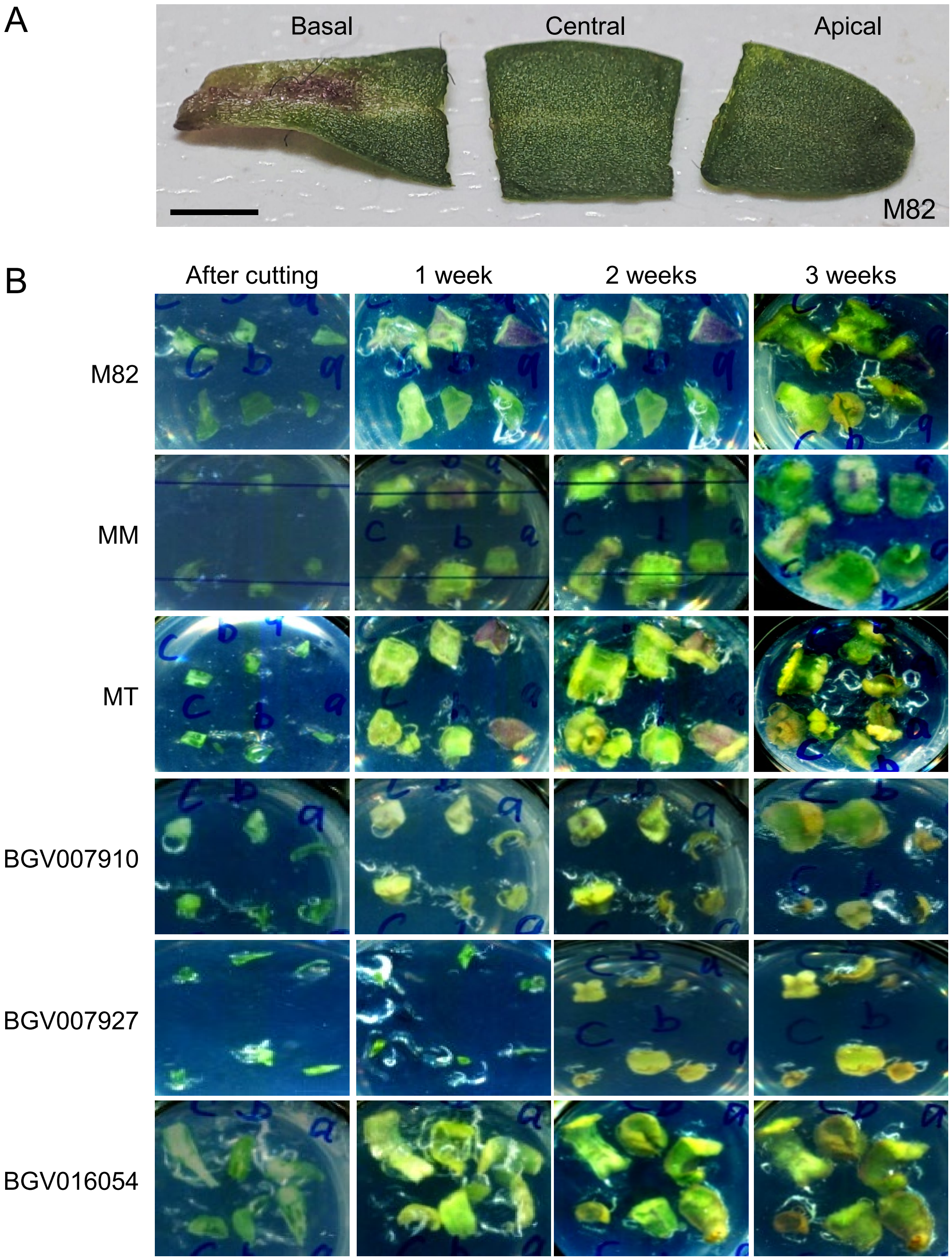

Suppl. Fig. S2. Experimental design to determine the effects of genotype and explant region on hormone-induced callus and shoot formation. (A) Apical, central, and basal regions of the cotyledon used as explants. M82 is shown as a representative example of the genotypes studied. (B) Representative images of explant incubation in supplemented medium for the six genotypes studied in this work. Scale bars: 3 mm..

Supplemental Figure S3

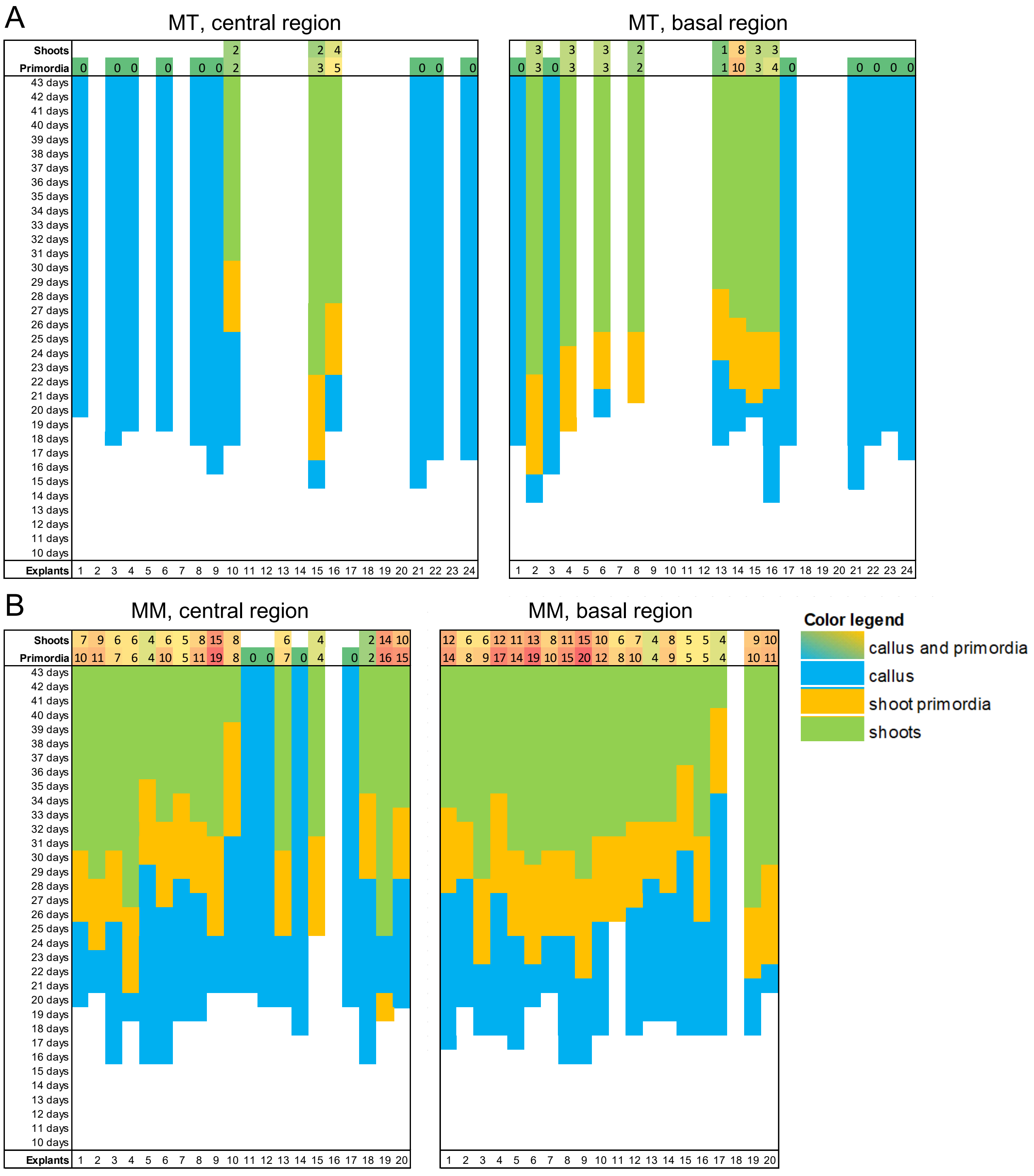

Suppl. Fig. S3. Timing of callus and shoot formation in individual tomato explants of MT (A) and MM (B).

Supplemental Figure S4

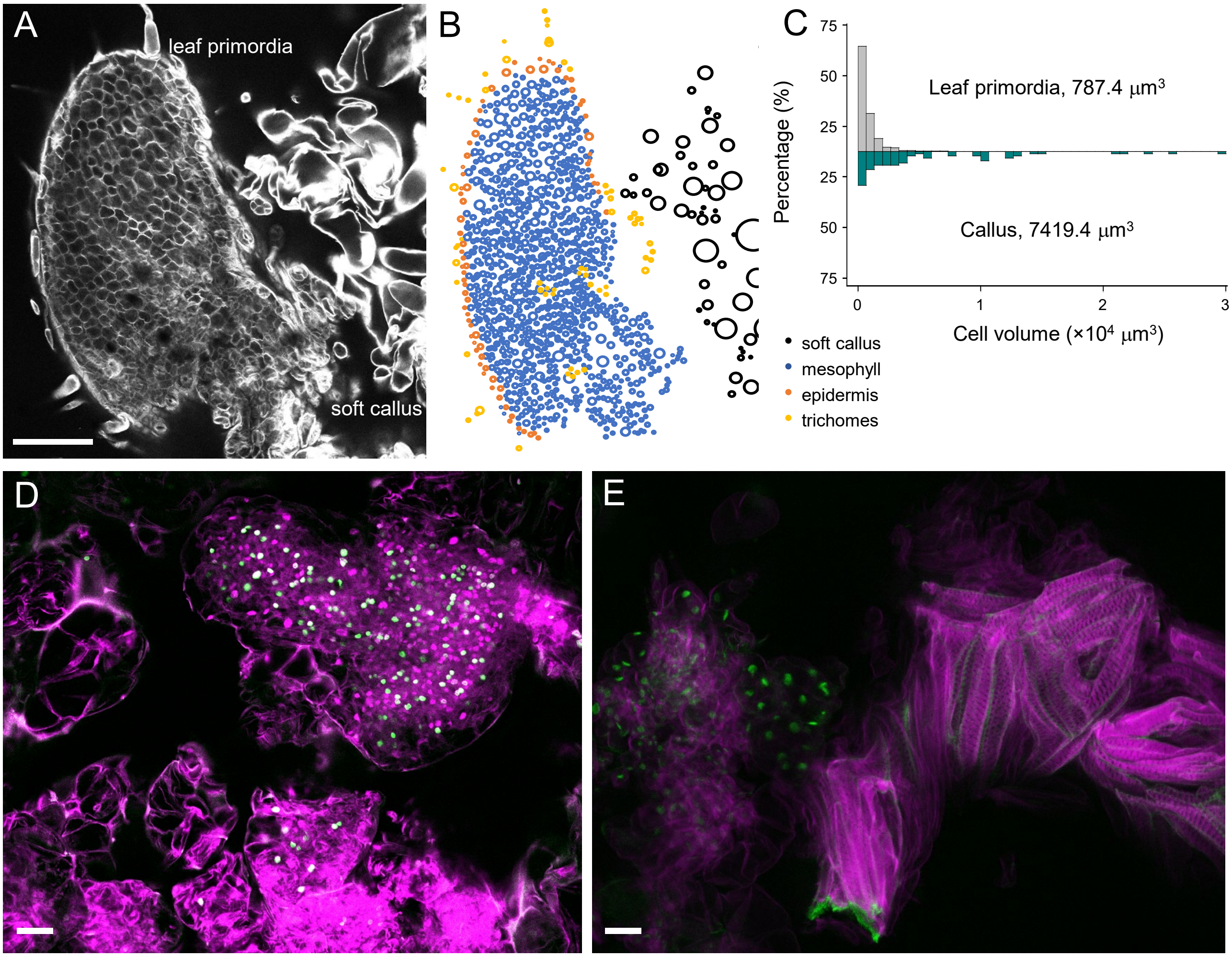

Suppl. Fig. S4. Cellular analysis of de novo leaf formation in tomato cotyledon explants. (A) A representative image of a leaf primordium and adjacent callus tissue in M82 cotyledon explants at 3 weeks. (B) Graphical representation of individual cell coordinates within the leaf primordium and adjacent callus indicated in B; bubble size represents cell volume. (C) Histogram of cell sizes from B. (D) Detailed observation of a globular callus tissue (green, 12 h EdU; magenta, SR 2200 cell wall staining) where de novo formed xylem cells are clearly visible (E). Scale bars: 40 µm.
